# Seagull: lasso, group lasso and sparse-group lasso regularization for linear regression models via proximal gradient descent

**DOI:** 10.1186/s12859-020-03725-w

**Published:** 2020-09-15

**Authors:** Jan Klosa, Noah Simon, Pål Olof Westermark, Volkmar Liebscher, Dörte Wittenburg

**Affiliations:** 1 Institute of Genetics and Biometry, Leibniz Institute for Farm Animal Biology, 18196 Dummerstorf, Germany; 2 Department of Biostatistics, University of Washington, Seattle, WA 98195 USA; 3 Institute of Mathematics and Computer Science, University of Greifswald, 17489 Greifswald, Germany

**Keywords:** Optimization, Machine learning, High-dimensional data, R package

## Abstract

**Background:**

Statistical analyses of biological problems in life sciences often lead to high-dimensional linear models. To solve the corresponding system of equations, penalization approaches are often the methods of choice. They are especially useful in case of multicollinearity, which appears if the number of explanatory variables exceeds the number of observations or for some biological reason. Then, the model goodness of fit is penalized by some suitable function of interest. Prominent examples are the lasso, group lasso and sparse-group lasso. Here, we offer a fast and numerically cheap implementation of these operators via proximal gradient descent. The grid search for the penalty parameter is realized by warm starts. The step size between consecutive iterations is determined with backtracking line search. Finally, *seagull* -the R package presented here- produces complete regularization paths.

**Results:**

Publicly available high-dimensional methylation data are used to compare *seagull* to the established R package *SGL*. The results of both packages enabled a precise prediction of biological age from DNA methylation status. But even though the results of *seagull* and *SGL* were very similar (*R*^2^ > 0.99), *seagull* computed the solution in a fraction of the time needed by *SGL*. Additionally, *seagull* enables the incorporation of weights for each penalized feature.

**Conclusions:**

The following operators for linear regression models are available in *seagull*: lasso, group lasso, sparse-group lasso and Integrative LASSO with Penalty Factors (IPF-lasso). Thus, *seagull* is a convenient envelope of lasso variants.

## Background

Linear regression is a widely used tool to explore the dependence between a response variable and explanatory variables. For example, in genome-wide association studies, counts of genetic variants along the genome are related to records of a disease or performance trait. The high throughput of modern biotechnological procedures enables studying an extremely large amount of explanatory variables (*p*). However, this often goes along with relatively few observations (*n*; *p* ≫ *n*), making the estimation of effects a challenge. As an example, high-dimensional methylation data are recently used to build regression models termed epigenetic clocks, which enable biological age to be predicted from DNA methylation status. Especially in the presence of multicollinearity, penalization methods have proved to be useful; Tikhonov, elastic net [1] and lasso [2] regularization are famous examples.

Standard approaches for epigenetic clocks employ elastic net regression, which performs well but typically results in only ~ 100 methylation sites with non-zero effect, limiting the potential for their genome-wide annotation and interpretation [3]. To simultaneously detect non-zero effects and account for the relatedness of explanatory variables, the lasso has been modified and enhanced to the group lasso [4], the sparse-group lasso [5] and the “Integrative LASSO with Penalty Factors” (IPF-lasso) [6]. These particular modifications of the lasso assume an underlying group structure within the explanatory variables. For instance, in genome-wide association studies, a group structure can be identified from linkage and linkage disequilibrium among chromosome regions. Thus, a method that exploits this structure such as the (sparse-)group lasso has the potential to improve the accuracy of results. *seagull* -the R package presented here- contains implementations of the lasso variants mentioned above focusing on precision of parameter estimation and computational efficiency.

Hereinafter we briefly describe the optimization problem and all relevant input parameters of *seagull*. We then use public data to evaluate our package and to compare it to the established R package *SGL* [7].

## Implementation

The R package *seagull* offers regularization paths for optimization problems of the form:

1 $$ \underset{\left(b,u\right)}{\mathit{\min}}\frac{1}{2n}{\left\Vert y- Xb- Zu\right\Vert}_2^2+\alpha \lambda {\left\Vert u\right\Vert}_1+\left(1-\alpha \right)\lambda {\left\Vert u\right\Vert}_{2,1}. $$

This is also known as the sparse-group lasso [5]. The first term expresses the “goodness of fit”. The second and third term are penalties, both of which are multiplied with the penalty parameter *λ* > 0. The vector *y* contains *n* observations of the response variable. The vectors *b* and *u* represent non-penalized and penalized effects, respectively; *X* and *Z* are the corresponding design matrices. Moreover, *α* ∈ [0, 1] is the mixing parameter which convexly links the penalties.

In the two limiting cases of *α* = 1 and *α* = 0, the resulting objective function is the lasso [2] and the group lasso [4], respectively. However, if *α* is chosen to be less than 1, it is assumed that the explanatory variables have an underlying group/cluster structure (with non-overlapping groups). Groups need to be determined prior to the call of *seagull*, for instance, by applying a suitable cluster algorithm to the explanatory variables or by grouping them according to the source of measurement (RNA expression, SNP genotypes, etc.). Referring to this structure, the entries of *u* can be separated into the corresponding groups, say *u*^(*l*)^ for group *l* and *p*_*l*_ is the size of group *l* (*L* is the total number of groups). Hence:

$$ {\left\Vert u\right\Vert}_{2,1}=\sum \limits_{l=1}^L\sqrt{p_l}{\left\Vert {u}^{(l)}\right\Vert}_2. $$

The penalty operators lasso, group lasso and sparse-group lasso are available in *seagull*. Furthermore, it is possible to consider weights for each explanatory variable and group. Thus, the implemented extension of the optimization problem (1) is:

2 $$ \underset{\left(b,u\right)}{\mathit{\min}}\frac{1}{2n}{\left\Vert y- Xb- Zu\right\Vert}_2^2+\alpha \lambda \sum \limits_{j=1}^p{\omega}_j^F\left|{u}_j\right|+\left(1-\alpha \right)\lambda \sum \limits_{l=1}^L{\omega}_l^G{\left\Vert {u}^{(l)}\right\Vert}_2, $$

where ${\omega}_j^F$ and ${\omega}_l^G$ are positive weights for feature *j* and group *l*, respectively. The weights for groups are defined as:

$$ {\omega}_l^G=\sqrt{p_l\overline{\omega_j^F}}, $$

where the average over weights of features is taken over those features that belong to group *l*, i.e., $\overline{\omega_j^F}=\frac{1}{p_l}{\sum}_{j\ \mathrm{in}\ \mathrm{group}\ l}{\omega}_j^F$. Hence, if all weights ${\omega}_j^F$ are set to 1, the optimization problem (2) yields problem (1).

The option of including weights can be used for any reason but it also enables the user to apply the strategy of IPF-lasso. In order to show this, we go back to optimization problem (2) with *α* = 1:

$$ \underset{\left(b,u\right)}{\min}\frac{1}{2n}{\left\Vert y- Xb- Zu\right\Vert}_2^2+\lambda \sum \limits_{j=1}^p{\omega}_j^F\left|{u}_j\right|. $$

For convenience, we assume the absence of any effects *b* and multiply the entire expression by 2*n*. Thus:

$$ \underset{u}{\min }{\left\Vert y- Zu\right\Vert}_2^2+2 n\lambda \sum \limits_{j=1}^p{\omega}_j^F\left|{u}_j\right|, $$

where a simplification can be obtained via ${\lambda}_j=2 n\lambda {\omega}_j^F$:

$$ \underset{u}{\min }{\left\Vert y- Zu\right\Vert}_2^2+\sum \limits_{j=1}^p{\lambda}_j\left|{u}_j\right|. $$

As a last step we assume that the entries of *u* are obtained from *M* different sources, i.e., “modalities” – as called by the authors of [6]. Then, we let all *λ*’s which belong to the same modality *m* have the same value *λ*^(*m*)^. Therefore, the last term in the above expression can be written as a sum over modalities:

$$ \sum \limits_{j=1}^p{\lambda}_j\left|{u}_j\right|=\sum \limits_{m=1}^M{\lambda}^{(m)}{\left\Vert {u}^{(m)}\right\Vert}_1. $$

And this immediately leads to the IPF-lasso. So in the *seagull* package, this particular lasso variant is implicitly included. The weights for features just need to be set accordingly, i.e., the same weight for features that belong to the same modality.

The penalty parameter *λ* > 0 reflects the strength of the penalization. Our package provides the opportunity to calculate a maximal value for *λ* (i.e., *λ*_*max*_) and to perform a grid search by gradually decreasing this value down to a minimal value (i.e., *λ*_*min*_). This minimum value is determined as a user-specified proportion *ξ* of *λ*_*max*_, i.e., *λ*_*min*_ = *ξλ*_*max*_. The sequence of penalty parameters is then calculated on a logarithmic scale. To efficiently accelerate the corresponding grid search, we implemented *warm starts*. Thus, the solution of *b* and *u* for the current value of *λ* is used as starting point for the subsequent value of *λ*. Eventually, *seagull* provides a sequence of penalty parameters and calculates the corresponding path of solutions.

The optimization problem is solved via *proximal gradient descent* (PGD; e.g., [8]). PGD is an extension of gradient descent for optimization problems which contain non-smooth parts, i.e., problems where the gradient is not available for the entire objective function. More details about this algorithm are presented in Additional file 4. As PGD is an iterative algorithm, a proper step size between consecutive iterations is crucial for convergence. This step size is determined with *backtracking line search*.

In the best case, an iterative algorithm such as PGD converges to the solution of the optimization problem. But typically, in the neighborhood of the solution the gain from one iteration to the next iteration decreases. Thus, a stopping criterion is implemented. Such a criterion is often based on a measurement of gain itself. In *seagull*, we implemented a stopping criterion which measures the gain from iteration *k* − 1 to *k* and scales it with the estimates at iteration *k*:

$$ \frac{{\left\Vert {\left(\hat{\begin{array}{c}b\\ {}u\end{array}}\right)}^{\left[k\right]}-{\left(\hat{\begin{array}{c}b\\ {}u\end{array}}\right)}^{\left[k-1\right]}\right\Vert}_{\infty }}{{\left\Vert {\left(\hat{\begin{array}{c}b\\ {}u\end{array}}\right)}^{\left[k\right]}\right\Vert}_2}\le {\varepsilon}_{rel}. $$

We refer to *ε*_*rel*_ as the relative accuracy, due to its definition as a ratio.

All implemented algorithms are based on the R package *Rcpp 1.0.3* [9].

## Data and evaluation criteria

We analyzed blood DNA methylation profiles at about 1.9 million CpG sites and its association with chronological age in mice (*n* = 141). The data set is publicly available and described in detail in [10]. We split the data set into training (*n* = 75) and validation (*n* = 66) data, where all age classes appeared almost equally in both sets, and applied the sparse-group lasso variant of *seagull 1.0.5*. R scripts for processing and analyzing the data are available in the supplementary material (Additional files 1 and 2). Ready-to-use data are also available at Code Ocean (see **Availability of data and materials**).

We compared the outcome of *seagull* to that of the established R package *SGL 1.3* [7]. Its implementation is based on accelerated generalized gradient descent. Both packages offer regularization paths to the same optimization problem (1). Thus, the input parameters for both packages are very similar. For example, we set the mixing parameter *α* to 0.95, a grid of 50 values for *λ*, and the ratio *ξ* between minimal and maximal *λ* equal to 0.001. However, despite the similarities between *seagull* and *SGL*, the implemented convergence criteria differ due to different meanings of accuracy parameters. In the *SGL* package, this parameter is an upper bound for the *ℓ*_1_-norm of the estimates of *b* and *u*. Unless stated otherwise, we set the accuracy parameter for *SGL* and *seagull* to 10^− 4^ and 10^− 6^, respectively.

We used the following criteria to evaluate the two packages: the minimum mean squared error (MSE) of predicted age based on methylation data (i.e., methylation age) and measured chronological age in the validation set along the regularization path, the squared correlation coefficient *R*^2^ between predicted and chronological age, the number of features with an estimated effect different from zero (i.e., non-zeros), and the execution time needed to compute the entire regularization path.

Another example for the application of *seagull* in genome-wide association studies is given in Additional file 3. It is shown how parameters (i.e., weights) can be tuned for IPF-lasso.

## Results and discussion

**Fig. 1:**
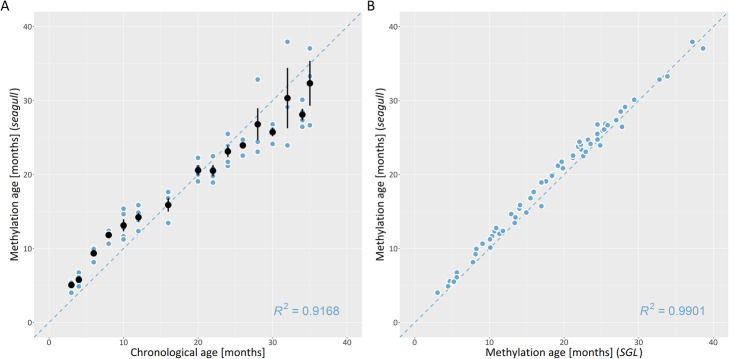
**a** Relationship between observed (chronological) and predicted (methylation) age. Each blue dot represents a sample in each class of observed chronological age (3mos, 4mos, etc.). Mean methylation age and error bars are displayed in black for each class of age. **b** Methylation age obtained with seagull vs. SGL. Blue dots represent samples; the dashed line is a regression line with slope 1

Figure 1a shows the model fit based on regression coefficients which led to the minimum mean squared error of chronological age in the validation set. The correlation between the chronological and the predicted age (“methylation age”) was 95.8%, and 5095 non-zero effects were identified with *seagull*. Hence, using only the identified fraction of CpG sites enabled a precise prediction of age. As an option for regulating the sparsity, increasing the accuracy parameter of *seagull* by two magnitudes (10^− 6^ to 10^− 4^) increased the number of non-zero effects by one magnitude. Though the implemented convergence criteria differed between both packages, results were similar. The correlation between regression coefficients leading to the minimum mean squared error was 99.5% (Fig. 1b). The number of non-zero effects obtained with *SGL* was 8822. In contrast to *SGL*, *seagull* computed the solution in a fraction of the time (*seagull*: ~ 2 h; *SGL*: ~ 45 h).

**Fig. 2:**
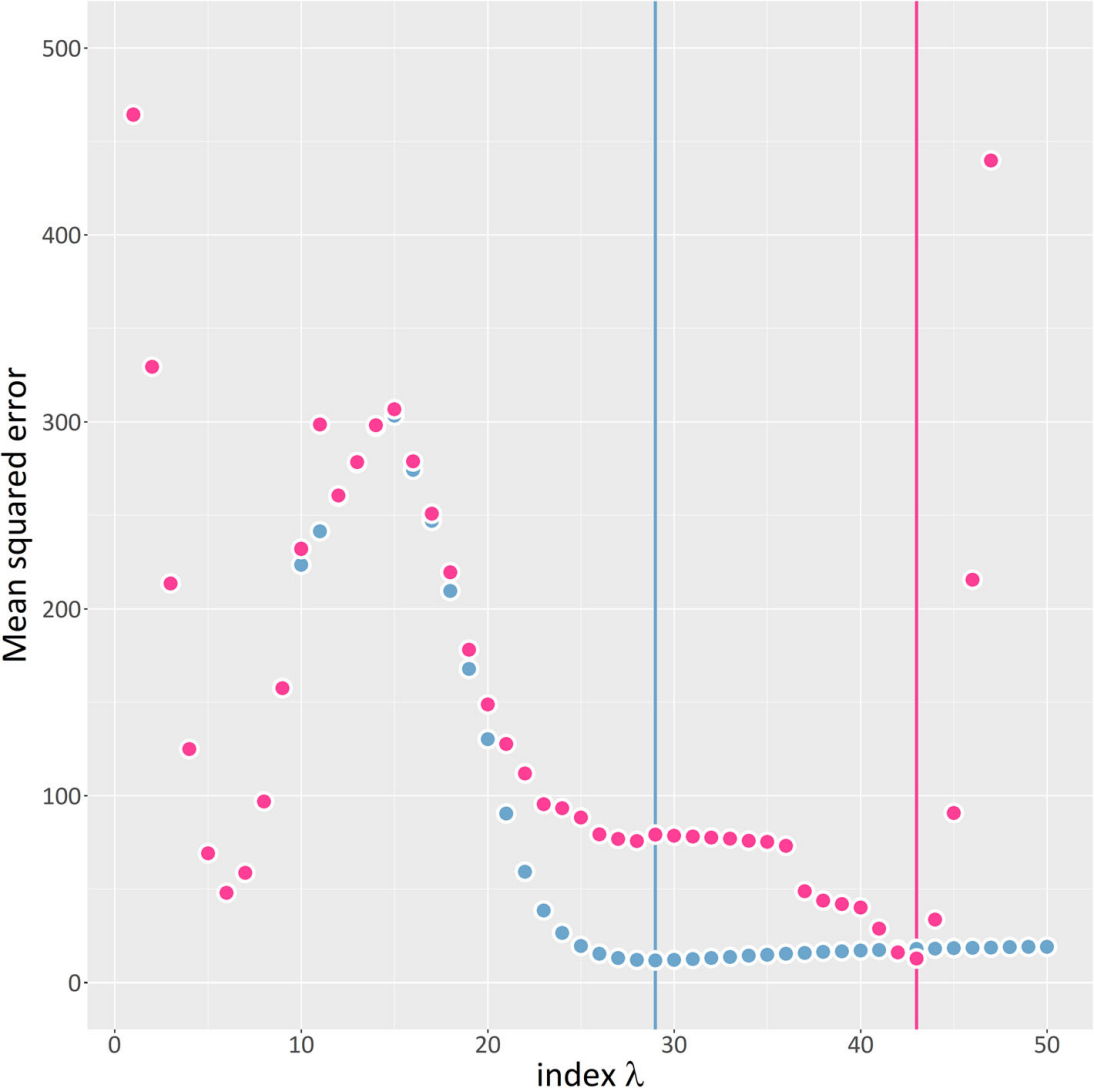
Path of mean squared error (MSE) of predicted age for each λ. Results of seagull and SGL are represented in blue and violet, respectively. The vertical lines mark the index in the sequence of λ’s with lowest MSE in corresponding color. The respective lowest MSE of seagull and SGL were 11.79 and 12.78

Table 1 displays the impact of the accuracy parameter on evaluation criteria in detail. If the accuracy parameter of *seagull* was set to 10^− 6^ or 10^− 8^, *seagull* outran *SGL* with respect to all evaluation criteria. The measures for *R*^2^ and non-zeros are both based on the value of *λ* for which the minimum MSE of prediction was obtained. The dependence between *λ* and the corresponding MSE is shown in Fig. 2.

**Table 1:** Performance evaluation

R package	Accuracy parameter	*R*^2^	MSE	Non-zero	Time
*SGL*	10^−4^	0.91	12.78	8822	45 h 20 min
*seagull*	10^−4^	0.92	12.38	65,463	20 min
*seagull*	10^−5^	0.92	11.57	11,823	40 min
*seagull*	10^−6^	0.92	11.79	5095	2 h 13 min
*seagull*	10^−8^	0.92	11.84	5072	4 h 50 min

Accuracy parameter refers to a package-dependent convergence parameter; *R*^2^ is the squared correlation coefficient and MSE is the mean squared error of chronological and predicted age; Non-zero denotes the number of CpG sites with non-zero effect estimate; Time is the computational time needed to calculate the full regularization path

In addition to *SGL*, *seagull* enables the opportunity to introduce weights for each penalized feature. This option was recently investigated in [11], where an optimization problem similar to (2) was used to estimate effects of SNP genotypes in a flowering plant breed (*Arabidopsis thaliana*). In that study, weights were defined according to the minor allele frequency (MAF) of genetic variants at each locus *j*, i.e., ${\omega}_j^F=2\sqrt{MAF_j\left(1-{MAF}_j\right)}$. Unlike *seagull*, the optimization problem described in [11] does not involve the incorporation of group weights other than the square root of the size of each group.

## Conclusions

Here we introduced our R package *seagull*, which offers regularization paths for the lasso, group lasso, sparse-group lasso, and IPF-lasso for linear regression models. We compared *seagull* to the established R package *SGL*. Both packages delivered similar results in terms of mean squared error, squared correlation coefficient, and sparsity pattern. Despite these similarities, *seagull* computed the solution in a fraction of time that *SGL* required. Furthermore, only *seagull* offered the opportunity to incorporate weights for each penalized variable which enables further variants of the lasso such as the IPF-lasso. In summary, *seagull* is a convenient envelope of lasso variants.

## Availability and requirements

**Project name:** seagull.

**Project home page:** https://CRAN.R-project.org/package=seagull

**Source code:** https://github.com/jklosa/seagull

**Operating system(s):** Platform independent.

**Programming language:** R, Rcpp.

**Other requirements:** R (> = 3.5.0).

**License:** GPL (> = 2).

**Any restrictions to use by non-academics:** None.

## Supplementary information

**Additional file 1.** An R script for downloading and processing the methylation data used in this study.

**Additional file 2.** An R script for the analysis of the processed data to generate Fig. 1.

**Additional file 3.** An R script for performing an exemplary genome-wide association study.

**Additional file 4.** A document with information about proximal gradient descent for the sparse-group lasso.

## Data Availability

The methylation dataset is available in the Gene Expression Omnibus (GEO) database, https://www.ncbi.nlm.nih.gov/geo/query/acc.cgi?acc=GSE80672. *seagull* is an R package that is freely available on the Comprehensive R Archive Network (CRAN; https://CRAN.R-project.org/package=seagull; vignette included). The source code is available on https://github.com/jklosa/seagull. The processed dataset, R scripts and results are also available at Code Ocean: https://codeocean.com/capsule/6412387/tree/v1.

## Abbreviations

IPF-lasso: Integrative LASSO with Penalty Factors
lasso: Least Absolute Shrinkage and Selection Operator
MAF: Minor Allele Frequency
MSE: Mean Squared Error
PGD: Proximal Gradient Descent
SNP: Single Nucleotide Polymorphism(s)
