# Supplementary material for: Seagull: lasso, group lasso and sparse-group lasso regularization for linear regression models via proximal gradient descent
Source: BMC Bioinformatics. 2020 Sep 15;21:407. doi: 10.1186/s12859-020-03725-w (PMC7493359; doi:10.1186/s12859-020-03725-w)
Supplement: Supplementary file 4 — Additional file 4. A document with information about proximal gradient descent for the sparse-group lasso. [file 12859_2020_3725_MOESM4_ESM.docx]

**seagull: lasso, group lasso and sparse-group lasso regularization for linear regression models via proximal gradient descent**

Jan Klosa^1^, Noah Simon^2^, Pål Olof Westermark^1^, Volkmar Liebscher^3^ and Dörte Wittenburg^1,*^

^1^Institute of Genetics and Biometry, Leibniz Institute for Farm Animal Biology, 18196 Dummerstorf, Germany

^2^Department of Biostatistics, University of Washington, Seattle, WA 98195, USA

^3^Institute of Mathematics and Computer Science, University of Greifswald, 17489 Greifswald, Germany

^*^Correspondence: [wittenburg@fbn-dummerstorf.de](mailto:wittenburg@fbn-dummerstorf.de)

**Additional file 4**

**Proximal gradient descent**

For more details about proximal methods and the required properties of the appearing functions $f$ and $g$, we refer to [1]. Consider the problem:

| $\mathrm{minimize} f\left( u \right)+g\left( u \right),$ | (1) |
| --- | --- |

where $f$ is convex and differentiable, and $g$ is convex. In the absence of the function $g$, one possible approach is the *gradient descent* algorithm, which starts with a guess $u_{0}$. Then, a sequence of iterates with step width $t>0$ is obtained via:

| $u_{m+1}= u_{m}-t\nabla f\left( u_{m} \right).$ | (2) |
| --- | --- |

However, in the presence of a non-differentiable function $g$ as in (1), the scheme (2) can be adapted in the following way:

| $u_{m+1}= \mathrm{prox}_{t}\left( u_{m}-t\nabla f\left( u_{m} \right) \right),$ | (3) |
| --- | --- |

where the operator $\mathrm{prox}_{t}$ is referred to as *proximal operator*. It is defined as:

| $\mathrm{prox}_{t}\left( r \right)= \underset{u}{arg min} \frac{1}{2t}\left\Vert r-u \right\Vert_{2}^{2}+g\left( u \right).$ | (4) |
| --- | --- |

Hence, the corresponding algorithm is:

| **given** $t>0$ and $u_{0}.$  **repeat**   1. Let $r:=u_{m}-t\nabla f\left( u_{m} \right).$ 2. Update ${u_{m+1}=prox}_{t}\left( r \right)= \underset{u}{arg min} \frac{1}{2t}\left\Vert r-u \right\Vert_{2}^{2}+g\left( u \right).$ | (5) |
| --- | --- |

This is called *proximal gradient descent*. The crucial step in this algorithm is that the proximal operator has a closed form. Perhaps somewhat surprisingly, whether or not this can be achieved relies solely on the function $g$.

Now, assume that we consider the following regression model:

| $y=Zu+e,$ | (6) |
| --- | --- |

where $e\in\mathbb{R}^{n}$ is a normally i.i.d. random variable. To solve this system of equations via the lasso, we aim to solve:

| $\mathrm{minimize}\frac{1}{2n}\left\Vert y-Zu \right\Vert_{2}^{2}+\lambda\left\Vert u \right\Vert_{1}.$ | (7) |
| --- | --- |

The first term is differentiable everywhere, whereas the second term is not differentiable in $0$. Therefore by comparison to (1), we identify the first term as $f$ and the second term as $g$. The proximal gradient descent formulation is then:

| **given** $t>0$ and $u_{0}.$  **repeat**   1. Let $r:=u_{m}-\frac{t}{n}Z^{T}\left( Zu_{m}-y \right).$ 2. Update $\forall j=1,\ldots,p: \left[ u_{m+1} \right]_{j}= \left\{ \begin{aligned} r_{j}-t\lambda, \mathrm{if} r_{j}>t\lambda\\ r_{j}+t\lambda, \mathrm{if} r_{j}<-t\lambda\\ 0 , \mathrm{else} \end{aligned} \right..$ | (8) |
| --- | --- |

The last expression is also called *soft thresholding* and is therefore also written using its own operator symbol $S$, i.e.:

| $u_{m+1}=S_{t\lambda}\left( r \right), \mathrm{with} \left[ S_{t\lambda}\left( r \right) \right]_{j}= \left\{ \begin{aligned} r_{j}-t\lambda, \mathrm{if} r_{j}>t\lambda\\ r_{j}+t\lambda, \mathrm{if} r_{j}<-t\lambda\\ 0 , \mathrm{else} \end{aligned} \right..$ | (9) |
| --- | --- |

As another example, we will write down the proximal operator for the sparse-group lasso, i.e., we consider the problem:

| $\mathrm{minimize}\frac{1}{2n}\left\Vert y-\sum_{l} Z^{\left( l \right)}u^{\left( l \right)} \right\Vert_{2}^{2}+\alpha\lambda\left\Vert u \right\Vert_{1}+\left( 1-\alpha\right)\lambda\sum_{l} \sqrt{p_{l}}\left\Vert u^{\left( l \right)} \right\Vert_{2}.$ | (10) |
| --- | --- |

Here the superscript $\left( l \right)$ denotes the $l$-th group. Again, the first term is differentiable everywhere, which is why we say this is the function $f$. The remaining two terms are not differentiable in $0$. Hence, now this is $g$. The proximal gradient descent formulation is then:

| **given** $t>0$ and $u_{0}.$  **repeat**   1. For each group $l$ let $r^{\left( l \right)}:=u_{m}^{\left( l \right)}-\frac{t}{n}Z^{\left( l \right)T}\left( Zu_{m}-y \right).$ 2. For each group $l$ update $u_{m+1}^{\left( l \right)}= \left( 1-\frac{\left( 1-\alpha\right)t\lambda\sqrt{p_{l}}}{\left\Vert S_{\alpha t\lambda}\left( r^{\left( l \right)} \right) \right\Vert_{2}} \right)_{+}S_{\alpha t\lambda}\left( r^{\left( l \right)} \right),$ | (11) |
| --- | --- |

where we used the soft-thresholding operator $S$ from the lasso. Furthermore, the + sign in the subscript indicates the positive part of the expression, i.e.:

| $\left( x \right)_{+}=\left\{ \begin{aligned} x , \mathrm{if} x>0 \\ 0, \mathrm{if} x\leq0 \end{aligned} \right..$ | (12) |
| --- | --- |

**Supplementary References**

1. Parikh N, Boyd S. Proximal Algorithms. FNT in Optimization. 2014;1(3):127–239.
